# Patterns of vestibular dysfunction in chronic traumatic brain injury

**DOI:** 10.3389/fneur.2022.942349

**Published:** 2022-12-01

**Authors:** Rachael L. Taylor, Kim J. Wise, Denise Taylor, Shikha Chaudhary, Peter R. Thorne

**Affiliations:** ^1^Department of Physiology, University of Auckland, Auckland, New Zealand; ^2^New Zealand Dizziness and Balance Centre, Auckland, New Zealand; ^3^Eisdell Moore Centre for Hearing and Balance Research, Auckland, New Zealand; ^4^Centre for Brain Research, University of Auckland, Auckland, New Zealand; ^5^Department of Psychology, University of Auckland, Auckland, New Zealand; ^6^Rehabilitation Innovation Centre, Health and Rehabilitation Research Institute, School of Clinical Science, Auckland University of Technology, Auckland, New Zealand; ^7^Section of Audiology, University of Auckland, Auckland, New Zealand

**Keywords:** concussion, traumatic brain injury, balance, vertigo, sensory integration, vestibular function

## Abstract

**Background:**

Dizziness and imbalance are common following traumatic brain injury (TBI). While these symptoms are often attributed to vestibular dysfunction, the relative contribution of peripheral vs. central mechanisms is unclear. This study investigated the prevalence of semicircular canal and otolith abnormalities in a cohort of patients with chronic TBI and symptoms of dizziness or imbalance. The relationship between vestibular, oculomotor and posturography results was further explored.

**Methods:**

Clinical records of patients attending the New Zealand Dizziness and Balance Centre from January 2015 to December 2019 were reviewed for consideration in the study. Inclusion required: an age of 18–80 years, a diagnosed TBI, and vestibular assessment using three-dimensional video head impulses (vHIT), cervical and ocular vestibular-evoked myogenic potentials (c and o VEMPs, respectively) and caloric testing. Severe TBI, pre-existing vestibular diagnoses, and incomplete test results were excluded. Rates of abnormalities were determined for each test and compared with results of oculomotor function testing and postural control, measured using the sensory organization test (SOT).

**Results:**

Of 158 reviewed records, 99 patients aged 49 ± 15 years (59 female) fulfilled criteria for inclusion in the study. The median time between the head injury and the clinical assessment was 12 (IQR 6–21) months. Abnormalities involving one or more components of the vestibular labyrinth and/or nerve divisions were identified in 33 of 99 patients (33.3%). The horizontal semicircular canal was most frequently affected (18.2%), followed by the saccule (14.1%), utricle (8.1%), posterior (7.1%) and anterior (2.0%) semicircular canals. Vestibular test abnormalities were associated with skull-base fractures, superior canal dehiscence, and focal ear trauma. Oculomotor dysfunction and postural instability were recorded in 41.1 and 75.5% of patients, respectively. Postural instability correlated with abnormal oculomotor function (*p* = 0.008) but not peripheral vestibular hypofunction (*p* = 0.336).

**Conclusions:**

Dizziness and/or imbalance in chronic TBI was associated with impaired postural stability for tasks requiring high levels of use of vestibular and visual input for balance. Vestibular hypofunction identified through vHIT, VEMP and caloric testing was recorded but was less common, except when the injury involved a fractured skull-base. There was no specific pattern of end-organ or nerve involvement which characterized this group of patients.

## Introduction

Traumatic Brain Injury (TBI) is estimated to affect 790 per 100,000 New Zealanders every year (1) and is a leading cause of disability in young adults. Symptoms of dizziness and imbalance are common and are among the risk factors predicting prolonged recovery (2, 3). These symptoms are often attributed to dysfunction of the vestibular system (4). However, the underlying mechanisms, and the relationship between vestibular function and performance on functional balance tasks, are poorly understood.

Vestibular dysfunction in chronic TBI may arise from trauma to the inner ear, or the brainstem, cerebellar and cortical networks involved in the processing of vestibular and other sensory inputs important for balance. Benign paroxysmal positional vertigo (BPPV), reported in up to 57% of TBI patients (4–7) is an example of a transient form of peripheral vestibular dysfunction, treatable with particle repositioning. Vestibular migraine, a central vestibular disorder, may also develop with or without associated vestibular test abnormalities (8). However, permanent loss of vestibular function, which we will refer to herein as peripheral vestibular hypofunction, may occur due to traumatic fracture or fistula of the bony and/or membranous labyrinth (4) or other microstructural injuries to the inner ear or vestibular afferents (9). Depending on the extent of central vestibular compensation, these injuries can lead to chronic symptoms. Reports of vestibular hypofunction are as high as 52% for symptomatic TBI patients (4, 7, 10–13). However, the prevalence and pattern/s of hypofunction are difficult to predict from most studies due to either small sample sizes (usually < 60), retrospective study design and/or incomplete evaluation of the five vestibular sensory organs.

Peripheral vestibular function can be topographically assessed using modern clinical tests. Among these tests are vestibular-evoked myogenic potentials (VEMPs) which enable assessment of the saccule (cervical VEMPs or cVEMPs) (14) and utricle (ocular VEMPs or oVEMPs) (15, 16), while video oculographic recordings during impulsive head turns applied in the plane of each semicircular canal provide the basis for video head impulse testing (vHIT) of semicircular canal function (17, 18). Abnormalities on oculomotor function tests complement the clinical examination to help differentiate between central and peripheral vestibular involvement. However, the assessment of higher-order vestibular processing, for example changes in cortical neuronal connectivity underlying vestibular self-motion perception or the gating and weighting of vestibular information, is more difficult due to the limited availability of perceptual and electrophysiological paradigms that can be performed outside of a research laboratory (19).

In this retrospective clinical case series, we report the results of vestibular function testing in a large group of TBI patients with ongoing symptoms of dizziness and/or imbalance. The aim of the study was to evaluate otolith and semicircular canal function using non-invasive, clinically applicable tests to: (i) determine how often, and which components of the peripheral vestibular system are affected; (ii) identify characteristics of the injury or clinical features that are associated with peripheral vestibular loss; (iii) explore the relationship between vestibular and oculomotor function and postural stability.

## Methods

### Patient selection and screening

The study was approved by the University of Auckland Human Participants Ethics Committee (Ref 024510) with a waiver for written consent. All patients were studied in accordance with clinical protocols as standard of care.

**Figure 1:**
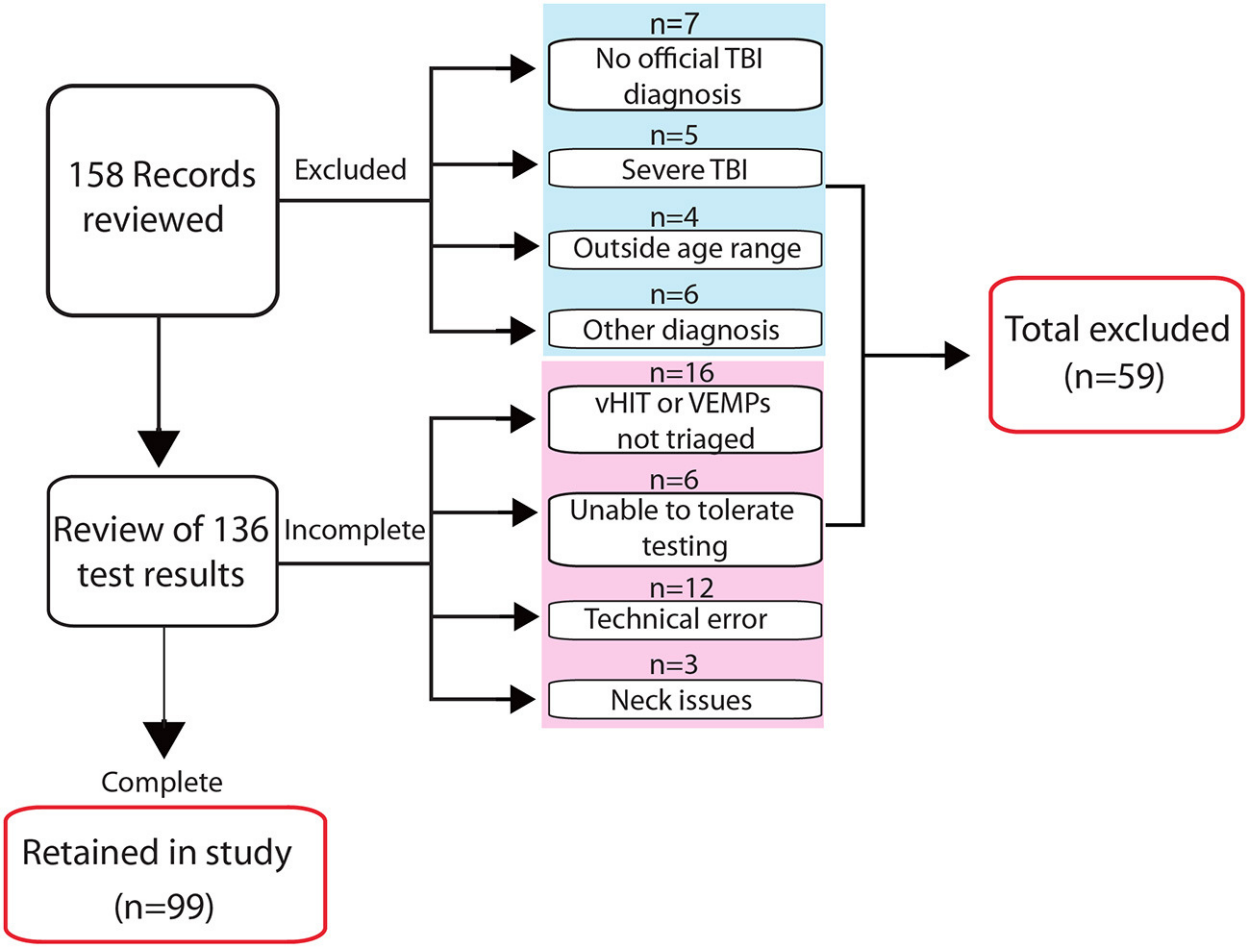
Screening process for inclusion in the study. The flow diagram indicates the number of records screened for inclusion in the study and reasons for exclusion at different stages in the review process. Four of five excluded patients classified in the referral notes as having a severe TBI had low Glasgow Coma Scale scores and/or prolonged periods of post-traumatic amnesia; the fifth documented “severe TBI” was without additional descriptive information.

Clinical records of individuals who attended the New Zealand Dizziness and Balance Centre three or more months following their injury, from January 2015 to December 2019, were reviewed for consideration in the study. Inclusion criteria required: an age of 18–80 years, a diagnosis of TBI/concussion from a medical practitioner, dizziness and/or balance symptoms where patients reported the onset following their injury, and a complete vestibular assessment using three-dimensional vHIT, cVEMPs, and oVEMPs. Excluded from further review were clinical referrals documenting severe brain injury, which in New Zealand is typically based on a Glasgow Coma Scale (GCS) of 3–8 and 7 days or more of post-traumatic amnesia (20), pre-existing vestibular or neurological diagnoses, and severe visual or musculoskeletal impairment (Figure 1).

Vestibular test results of individuals meeting the inclusion criteria were reviewed for rates of abnormalities referrable to each sensory organ. Caloric, oculomotor function and posturography results were analyzed where data were available and compared with other clinical information. Additional assessments such as the motor control test, dynamic visual acuity, audiometry, functional gait assessment and other bedside examinations were variably undertaken or were performed by another service and thus, results were not always available for review and so were not included. Further, although headaches and migraine were common in this cohort, the exact number of “episodes” of vestibular symptoms and the headache characteristics, both requirements for a diagnosis of vestibular migraine (21), was not always documented and so the prevalence of vestibular migraine was not explored.

### Test procedures

#### Video head impulse testing of semicircular canal function

An ICS Impulse (GN Otometrics, Tastraap, Denmark) vHIT system recorded head and eye velocity in response to small, unpredictable head movements delivered in the plane of each semicircular canal. Raw data were reviewed using version 4.1 analysis software prior to inclusion in the study; traces containing intrusive artifact were manually deleted. To minimize errors in classification due to technical error on vertical canal testing (22), an abnormal response required both a low mean gain (<0.8 and <0.7 for the horizontal and vertical canals, respectively) and repeatable refixation saccades.

#### Vestibular evoked myogenic potential tests of otolith function

Vestibular evoked myogenic potentials (VEMPs) were recorded using an ICS Chartr^®^ EP 200 system (Natus Medical). Detailed methods are provided in Supplementary material 1. Briefly, cervical VEMPs (cVEMPs) were measured from the ipsilateral sternocleidomastoid muscle in response to 500 Hz air-conducted tone-bursts. Ocular VEMPs (oVEMPs) were measured infra-orbitally from the contralateral eye during maximum upward gaze to 500 Hz bone-conducted tone-bursts delivered *via* a hand-held minishaker (model 4810, Bruel & Kjaer) to the upper forehead, near the hairline. Responses to two trials of stimuli (125 for cVEMPs; 60 for oVEMPs) presented at 5 Hz were recorded and averaged. Absent or asymmetrical responses, determined from the Jongkees formula (23) by an asymmetry ratio (AR) > 30% (cVEMP) and 40% (oVEMP), were considered abnormal as per clinic protocol. Patients with middle ear pathology or aged over 60 years, had additional cVEMP testing with bone-conduction stimulation before classifying a response as abnormal. Asymmetry calculations for cVEMPs were based on corrected (normalized) amplitudes to account for differences in tonic muscle activation.

#### Caloric and oculomotor function assessment

Videonystagmography (ICS Chartr VNG 200, version 6.2.0; GN Otometrics) quantified eye movements during caloric and oculomotor function testing. The protocol included gaze holding (center and 30° left and right) with and without visual fixation, horizontal saccades, pursuit, positional tests and VOR suppression. Caloric testing involved air irrigated at 24° and 50° Celsius for 60 s according to previously published protocols (24). In the clinic, unilateral weakness was interpreted from an asymmetry ratio > 25% (25) based on manufacturer default settings; responses were considered bilaterally weak (abnormal) if the sum of the maximum nystagmus slow-phase velocity for all four irrigations was below 20°/s. Results involving reduced irrigation times or a monothermal protocol (due to heightened autonomic responses and/or motion sensitivity) were retained in the study provided the asymmetry was < 15%, as per published recommendations (26). Oculomotor function results were interpreted from review of the clinic notes, visual inspection of the eye position traces, and comparison of quantitative outcome measures; namely saccade accuracy, velocity and latency and smooth pursuit velocity gain (0.1–0.5 Hz), against manufacturer-specified, normal age-referenced ranges.

#### Postural stability using computerized dynamic posturography

The Sensory Organization Test (SOT) evaluated the use of sensory information for balance (NeuroCom^®^ International Inc, version 9.0). Testing involved manipulation of the support surface (stable/ sway referenced) and vision (eyes open/eyes closed/ sway referenced), giving six conditions (C 1-6, Table 1).

**Table 1:**
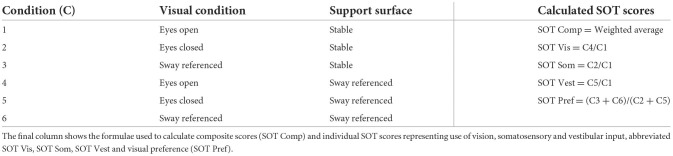
Conditions for the sensory organization test.

The final column shows the formulae used to calculate composite scores (SOT Comp) and individual SOT scores representing use of vision, somatosensory and vestibular input, abbreviated SOT Vis, SOT Som, SOT Vest and visual preference (SOT Pref).

Three trials of 20 s were recorded to generate a score from 0 to 100 for each condition, where 100 = no sway/minimal sway and 0 = fall/near fall. The analysis software produced a composite score, a weighted average for the six conditions, and ratio scores indicating how much manipulation of the support surface and vision affected the scores. A visual preference score was also calculated which is suggested to estimate the ability of the individual to ignore incorrect visual input. Scores were compared with manufacturer-specific, age-referenced 95 percentile limits of normal (27).

#### Statistical analysis

Data were analyzed using SPSS (IBM, version 28). Demographic features and test abnormalities are reported as frequencies for categorical variables and median (range) or mean (SD) for continuous data. Binary logistic regression was used to investigate the relationship between skull-base fractures (SBF) and vestibular hypofunction, while controlling for age, sex, and causes of injury in the model. The relationship between SOT abnormalities, which are already age-referenced, and abnormalities on tests of peripheral vestibular dysfunction (Yes/No) and central oculomotor function (Yes/No) was explored using the same model but excluding age. The relationship between continuous outcome measures, some of which were not normally distributed, was explored using appropriate non-parametric methods. Significance was set at 5% (*p* < 0.05) after adjustment for multiple comparisons (Bonferroni-Holm).

## Results

**Figure 2:**
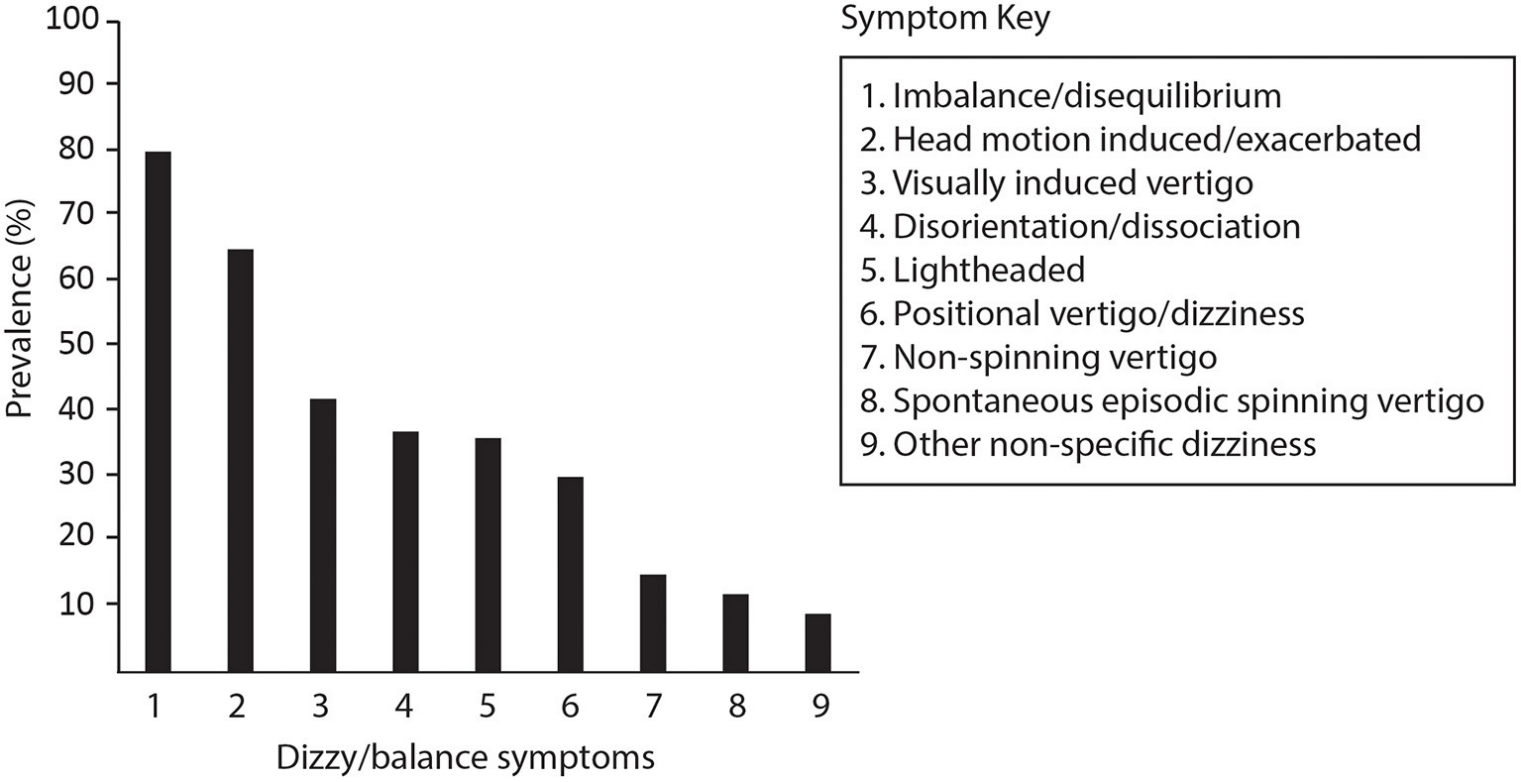
Symptoms reported by patients at the time of their vestibular assessment. Imbalance/disequilibrium includes self-reported balance difficulties of any degree. Head-motion induced symptoms refer to visual disturbance, imbalance, vertigo or dizziness and nausea specifically provoked or exacerbated by head movements during usual daily activities. Non-spinning vertigo included sensations of self-motion other than spinning such as tilting, rocking, swaying, falling, or “like being on a boat” which were either constant or episodic. Episodic spontaneous spinning vertigo refers to at least two episodes of spinning sensations lasting minutes to hours without a precipitating trigger. Non-specific dizziness was reserved for other descriptions and cases where no other description was provided.

Of 158 reviewed records, 99 symptomatic patients aged 49 ± 15 years (59 female) fulfilled the criteria for inclusion in the study. Referrals came from community concussion clinics, neurologists, ENT specialists, general practitioners and physiotherapists. Falls^1^ (36.4%), sporting injuries (19.2%), motor vehicle accidents (17.2%) and assault (5.1%) were the most common causes of TBI. Review of the clinical notes indicated that patients usually reported more than one type of dizzy/balance symptom (Figure 2), attributing the onset of at least one symptom to the time of their injury. While some were able pinpoint the onset as immediately after the injury or after leaving hospital, others were not and could only say they became aware of symptoms soon after the injury.

The median time from the injury to the clinical assessment was 12 months (IQR = 6–21 months; min-max = 3–120). Most (95) TBI had been classified by the referring physician or another involved physician as “mild (mTBI)” or “concussion”; four were moderate. However, while it is usual to classify severity according to GCS and post-traumatic amnesia, only 12 referrals disclosed the actual GCS score. Eleven of 99 injuries were associated with an SBF; including one moderate TBI.

### Patterns of peripheral vestibular dysfunction

In addition to vHIT and VEMP tests, 82 patients had caloric results which were included in the review. The main reasons for missing caloric data were presence of outer/middle ear pathology (*n* = 7), test intolerance (*n* = 5), test not requested/triaged (*n* = 3), technical problems (*n* = 2).

Of the 99 reviewed cases, 33 (33.3%) had abnormalities involving one or more vestibular organs/afferent divisions. Patients with and without vestibular test abnormalities did not differ significantly, either in age (52 vs. 47.1 years, respectively, *p* = 0.121) or median time from the injury to the clinical assessment (12 vs. 11 months, respectively, *p* = 0.402), although the inter-quartile range was larger for the group with hypofunction (6–30 vs. 6–19 months). Falls as a cause of TBI were more common in the group with vestibular hypofunction (54.5 vs. 27.3%). However, the association was not significant after controlling for age, sex and SBF (*p* = 0.485).

Table 2 shows diverse patterns of dysfunction. The horizontal semicircular canal (HC) was most frequently affected with 18 of 99 cases (18.2%) identified through combined assessment using vHIT and caloric testing. The caloric test had a slightly higher yield than vHIT and two patients with dissociated results on these tests (abnormal caloric and normal vHIT) presented with recurrent episodic vertigo lasting hours. Abnormalities were also found in the function of the saccule (14.1%), the utricle (8.1%), the posterior (7.1%), and anterior (2.0%) canals (PC and AC, respectively). Deficits were unilateral in 29 cases. Three patients showed bilateral vHIT abnormalities involving the HC (*n* = 2) or PC (*n* = 1); a fourth patient had an abnormal *left* PC with an impaired *right* saccule.

**Table 2:** Profiles of test results for patients with vestibular hypofunction.

**Injury details**				**Vestibular function testing**									**Posturography**			
	**Injury type**	**CT imaging**	**BPPV**	**Caloric** **(HC)**	**HC vHIT**		**AC vHIT**		**PC vHIT**		**cVEMP** **(saccule)**	**oVEMP** **(utricle)**	**SOT** **Comp**	**SOT** **Vest**	**SOT** **Vis**	**SOT Som**
					**R**	**L**	**R**	**L**	**R**	**L**						
1	SRC	SBF L	+/–	−54% (L)	0.90	0.83	0.68*	0.91	0.94	0.63*	7.6%	15.1	72	59	69	99
2	Fall	SBF R	–/–	37% (R)	1.01	0.91	0.90	0.83	0.42	0.85	42% (R)	4.0%	69	52	75	91
3	Fall	SBF L	+/+	−15%	0.90	0.75	0.91	0.81	0.74	0.84	−100% (L)	−28.7%	75	60	86	96
4	MVA	SBF L	+/–	−37% (L)	1.02	0.95	0.91	0.89	0.79	0.93	−25%	22.9%	44	0	53	92
5	Fall	SBF R	–/–	100%(R)	0.30	0.80	0.40	0.50*	0.37	0.94	100%(R)^†^	100% (R)	45	35	63	95
6	MVA	SBF R+L	–/–	14%	0.98	0.89	0.94	0.85	0.68	0.89	−5%^†^	12.3%	67	57	78	99
7	Fall	SBF L	–/–	19%	0.70	0.73	0.51*	0.55*	0.68*	0.53*	10.8%	57% (R)	56	41	52	96
8	Fall	SBF R	–/–	−10%	1.09	0.92	1.16	0.70	0.82	1.00	45.5% (R)	11.9%	41	0	56	92
9	Fall	SBF R	+/+	75% (R)	1.01	0.96	0.82	1.28	0.83	0.79	100% (R)	2.7%	53	0	70	76
10	Fall	SCD R	–/–	NA	1.02	0.94	0.42	0.97	0.70	0.93	−40.1% (R)	−89% (R)	NA	NA	NA	NA
11	Other		–/–	32% (R)	0.79	0.78	0.89	1.03	0.81	0.78	32% (R)	−6.0%	77	69	91	98
12	Fall		+/–	91% (R)	0.94	0.97	0.77	0.92	0.87	0.78	−21.5%	−2.0%	40	0	70	89
13	Other		–/–	18%	1.08	0.96	0.87	0.92	0.81	0.97	56% (R)	−21%	74	58	83	96
14	Fall		–/–	9%	0.99	0.92	1.07	1.23	1.11	0.65	58.1%(R)	0%	64	29	82	91
15	SRC		–/–	−4%	0.93	0.54	0.87	0.74	1.01	0.80	−11.5%	20.8%	60	44	73	90
16	MVA		–/–	NA	1.00	0.87	0.86	0.68*	0.67*	0.78	−5.8%	70.5% (R)	35	0	39	80
17	Fall		+/+	−56% (L)	1.39	1.10	1.09	0.90	0.95	1.03	−16.4%	−31.9%	66	51	86	98
18	Fall		–/–	52% (R)	0.97	0.94	0.77	0.74	0.75	0.88	6.0%	12.0%	20	0	29	22
19	Fall		–/–	NA	1.13	1.06	0.85	0.97	0.72	0.66	−33.0% (L)^†^	22.0%	36	0	46	90
20	MVA		–/–	0%	1.14	1.07	0.73	0.95	0.90	0.69*	−6.0%	48.6% (R)	50	34	74	95
21	Other		–/–	3%	1.12	1.08	1.02	0.99	0.85	0.85	−100% (R)	1.8%	51	41	63	93
22	Fall		–/–	−45% (L)	0.99	0.96	0.91	0.89	0.79	0.93	−1.0%	27.8%	76	29	95	95
23	MVA		–/–	18%	1.00	0.96	0.94	0.57*	0.53*	0.82	10.9%	−43.8% (L)	70	73	76	93
24	Fall		–/–	NA	0.92	0.68	0.81	0.76	0.37	0.39	−17.6%	−2.6%	10	0	0	65
25	Fall		–/–	−40% (L)	0.95	0.70	0.97	0.89	0.89	0.95	6.0%	5.4%	66	65	83	98
26	Other		–/–	30% (R)	0.98	0.92	0.90	0.81	0.80	0.75	6.6%	−4.0%	77	67	88	94
27	Fall		+/+	50% (R)	0.99	0.95	0.86	0.61*	0.65*	0.92	−8.4%	2.8%	69	65	82	96
28	Fall		–/–	−18%	1.10	0.94	0.97	0.96	0.86	0.84	−55% (L)^†^	21.1%	69	39	81	93
29	Fall		–/–	NA	0.91	0.85	0.88	0.81	0.66	0.73	4.7%	20.1%	NA	NA	NA	NA
30	MVA		–/–	−18%	1.14	1.01	0.94	1.06	1.14	0.97	42.2% (R)	24.5%	79	75	88	93
31	SRC		–/–	6%	0.97	0.94	0.63*	0.72	0.74	0.81	−52.2% (L)^†^	−61.6% (L)	42	0	63	82
32	Other		–/–	−32% (L)	1.00	0.85	1.07	1.08	0.94	0.94	2.5%	7.2%	52	37	58	75
33	Assault		–/–	9%	1.12	1.00	0.97	0.78	0.79	1.03	6.1%	49.4% (R)	32	16	54	70
Total abnormalities			4	14	7		2		7		14	8	21	21	18	8

Vestibular function tests and SOT results are classified as within normal limits, abnormal or not tested/analyzed (NA). Abnormalities on assessment are highlighted in gray. Asymmetry ratios for caloric and vestibular evoked myogenic potentials (VEMPs) with a negative sign (–) represent a weaker response from the left ear; affected ears are indicated in parentheses (VEMP and caloric), abbreviated L and R for left and right. Benign paroxysmal positional vertigo, BPPV (+/+) = previously treated/present at time of assessment. Abbreviations for imaging and injury details: SBF, Skull-base fracture; SCD, superior canal dehiscence; SRC, sports-related concussion; MVA, motor vehicle accident. Asterisks indicate vertical canal head impulses with gain below 0.70 but no refixation saccades (considered normal in this study);

† indicates cases where cVEMPs were interpreted from bone conduction results due to bilateral or unilateral middle ear pathology (*n* = 4) or loudness sound sensitivity (*n* = 1). Total abnormalities represent the number of patients with an abnormal result on each test.

#### Skull base fracture and other fistulae

**Figure 3:**
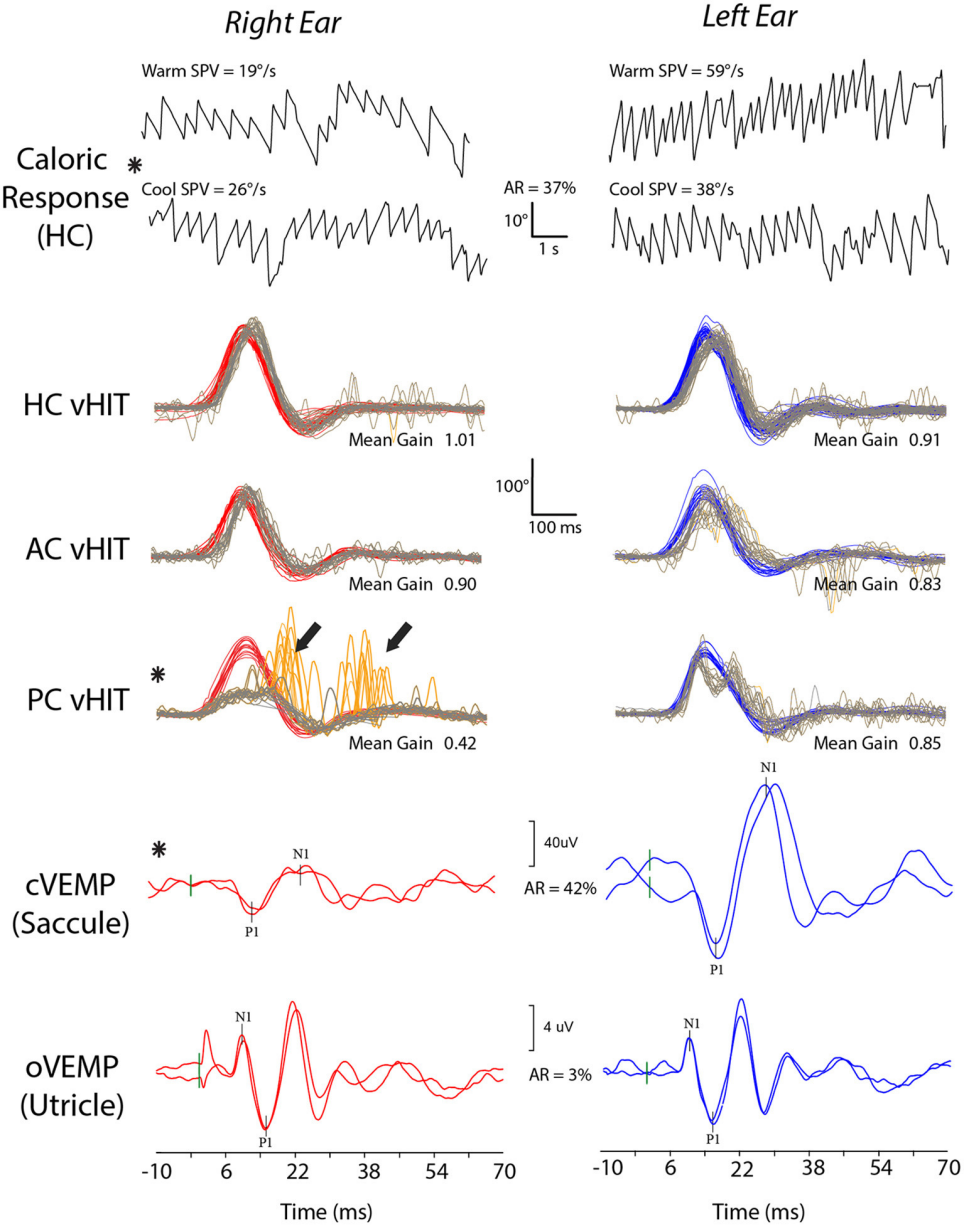
Vestibular test results for a patient with TBI who sustained a right skull-base fracture. The patient shows a right vestibular weakness with a 37% right horizontal canal (HC) paresis on caloric testing and reduced function of the right saccule (asymmetry ratio, AR, of 42%) and posterior canal (PC). Abnormal test results are indicated by asterisks. Bold arrows indicate large catch-up saccades on posterior canal video head impulse testing (vHIT).

There was a significant relationship between the presence of abnormalities on vestibular function tests and SBF (*X*^2^ = 6.200; *p* < 0.013) but not age or sex (*p* = 0.863 and 0.560). Compared to TBI without SBF, the odds of vestibular hypofunction were 8.5 (CI: 1.6–45.8) with 9 of 11 patients with SBF (81.8%) demonstrating abnormalities referrable to one or more vestibular organs. New onset hearing loss in the same ear as the vestibular deficit was documented in all but one of these cases. Other associated findings in SBF were: perilymph (round window) fistula; facial nerve weakness; mastoid effusions; middle ear pathology (tympanic membrane perforation, ossicular chain dislocation, recurrent effusion). Vestibular test results for a patient with a right SBF are shown in Figure 3.

One patient had radiological evidence of superior semicircular canal dehiscence (SCD) and asymmetrical oVEMP amplitudes due to an enlarged response from the affected ear. The patient also had an AC deficit on vHIT, reduced cVEMP thresholds and torsional nystagmus to vertex vibration.

#### Benign paroxysmal positional vertigo

Nine patients tested positive for BPPV when seen for their vestibular and balance assessment 3–11 months after their TBI, all with nystagmus profiles consistent with posterior canalithiasis. Of these, eight had been experiencing recurrent positional vertigo beginning within a month of their injury with variable responses to previous attempts at particle repositioning. Twenty patients who tested negative at the time of their assessment were documented as having received treatment for BPPV since their injury.

### Oculomotor function

Abnormalities on one or more tests of central oculomotor function were recorded in 39 of 95 cases (41.1%) with available data. Abnormal smooth pursuit characterized by low gain was the most common abnormality (23.4%), followed by prolonged saccade latencies (18.9%), slower than normal saccade velocities (12.6%), bidirectional gaze-evoked or vertical nystagmus in darkness (i.e., without visual fixation) (6%), poor VOR suppression (3.6%) and inaccurate saccades (3.2%).

No nystagmus was recorded with visual fixation. Thirty six of 90 patients (40%) with interpretable results had low velocity nystagmus that was horizontal and unidirectional in one or more gaze positions in darkness (Median peak SPV = 2°/s; range = 0.5–4°/s). In patients with vestibular hypofunction (*n* = 18), both ipsilesional and contralesional quick phases were recorded. In all cases, the SPV fell within the range reported in healthy controls (28) and was not analyzed further.

### Postural stability

Ninety-six patients had posturography results available for review; two were excluded due to inconsistent/uninterpretable or incomplete results, leaving 94 sets of results for analysis. Some SOT scores were not normally distributed due to high numbers of loss of balance classified as a “fall” by the software (scores of 0) on C5 indicating poor balance in conditions that require high use of vestibular information (*n* = 23); scores of 0 on C2 (stable support, eyes closed) and C4 (unstable support, eyes open) were documented in six and three cases, respectively.

Seventy one of 94 patients (75.5%) had abnormal results on one or more SOT scores. Deficits in balance during tasks that required significant use of vestibular, visual and somatosensory input were identified in 66, 55.3, and 27.7% of patients, respectively, with 47 patients (50.0%) demonstrating problems across multiple sensory domains. Difficulty in ignoring incorrect visual information (indication of possible over-reliance on vision for balance) was seen in 25 patients (26.6%). A breakdown of the different combinations of abnormal SOT scores for the three sensory inputs is provided in Table 3.

**Table 3:** Patterns of abnormalities on the sensory organization test.

**Multi-sensory impairment**	***n*** **=** **47**
Vision, vestibular and somatosensory	*n* = 25
Vision and vestibular	*n* = 21
Vestibular and somatosensory	*n* = 1
Vision and somatosensory	*n* = 0
**Single-sensory impairment**	***n*** **=** 21*
Vestibular	*n* = 15
Vision	*n* = 6
Somatosensory	*n* = 0

* An additional four patients with normal scores for each individual sensory condition had only abnormal visual preference.

**Figure 4:**
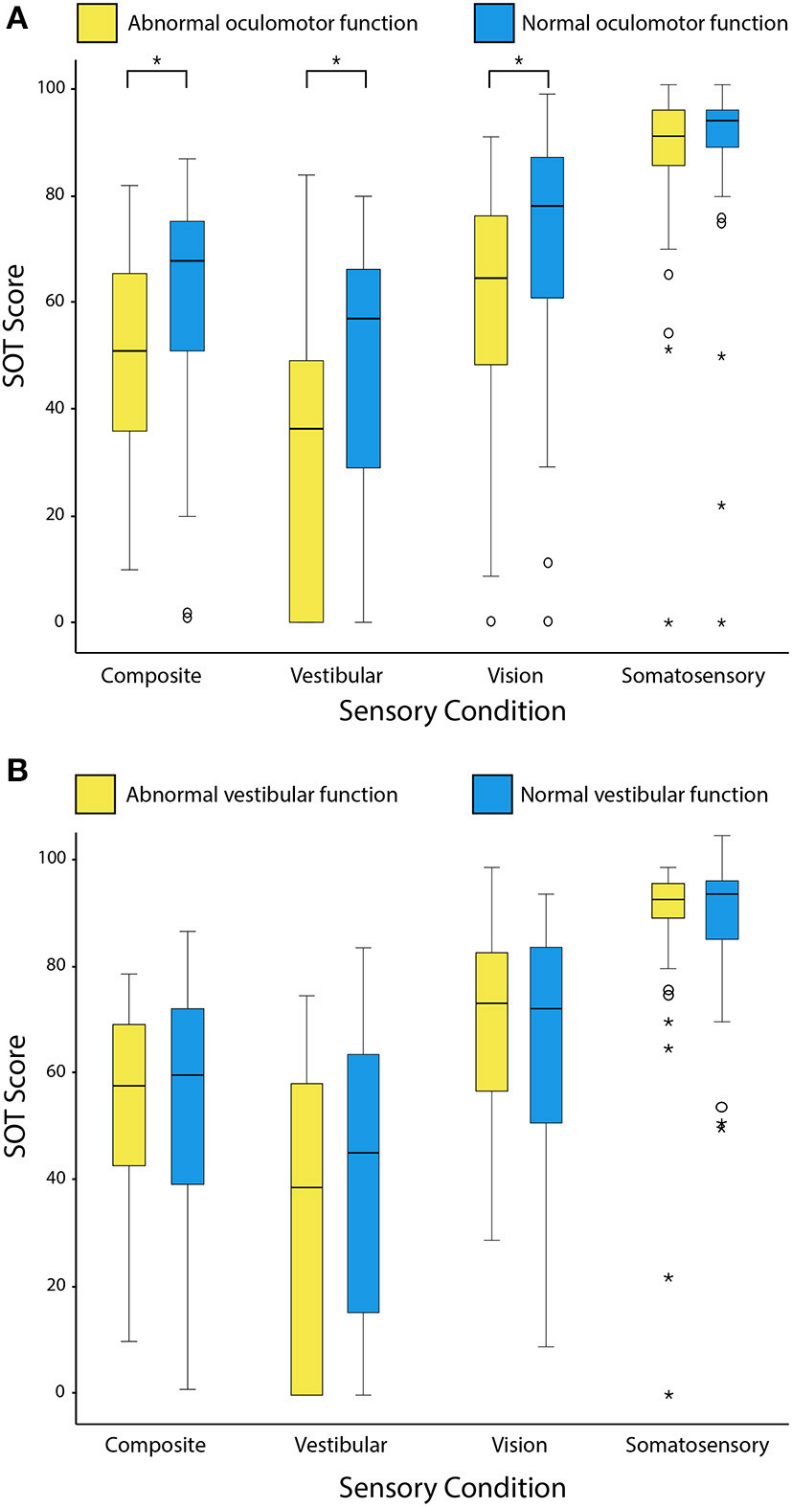
Box and whisker plots comparing the distribution of raw SOT scores grouped according to the presence or absence of oculomotor dysfunction **(A)** and peripheral vestibular hypofunction **(B)**. Outlying values are indicated by open circles and asterisks. Composite scores are the weighted average of all six SOT conditions. Scores indicating use of sensory information (vestibular, vision and somatosensory) are a ratio of the respective individual conditions (C5, C4, and C2) to the baseline condition (C1). Higher scores indicate better postural stability and better use of sensory information for balance. Asterisks indicate adjusted *p*-values < 0.05 on Mann-Whitney U tests.

Figure 4 compares the raw SOT scores of TBI patients grouped according to presence or absence of an oculomotor abnormality (A) and a peripheral vestibular deficit (B). Logistic regression confirmed a significant relationship between the presence of central oculomotor dysfunction and abnormal postural sway on composite SOT scores (adjusted *p* = 0.008; OR = 6.4; 95% CI: 1.9–21.2). Patients with oculomotor dysfunction also had greater difficulty using vestibular input for balance (adjusted *p* = 0.005; OR = 7.7; 95% CI: 2.2, 27.0). A peripheral vestibular deficit was not a significant predictor of an abnormal composite score (*p* = 0.336) or any of the individual sensory scores (*p*-value range: 0.386–0.972).

## Discussion

### Prevalence and patterns of peripheral vestibular hypofunction

Few studies have reported on the functional evaluations of each of the five vestibular organs in the same patient with chronic TBI. Our testing identified vestibular hypofunction in a third of patients, with increased likelihood for those with a skull-base fracture. Abnormalities were usually unilateral and restricted to only one or two sensory organs/afferent nerve divisions.

Previous studies of vestibular function in chronic TBI have relied mainly on caloric testing and, more recently, horizontal vHIT. Consistent with our findings, abnormality rates on either test are often < 25% with few reports of bilateral involvement (4, 12, 13, 29–31). Three studies which included vertical canal vHIT showed increased detection rates with reports of abnormal function of at least one semicircular canal in 21.2–52% of patients (12, 32, 33). Otolith function has been mainly studied using cVEMPs with rates of saccular dysfunction ranging between 0 and 52% (7, 12, 13, 29, 31, 33–35). Use of oVEMPs to test utricular dysfunction in TBI has rarely been reported. Campbell et al. described asymmetrical oVEMPs in five of 29 (17%) of patients with chronic TBI, which was not significantly different from age-matched controls (31), whereas Misale et al. reported abnormalities in 18 of 36 patients (50%) with TBI and non-positional, post-traumatic episodic vertigo (33).

There was no consistent pattern of sensory organ and/or nerve involvement in our case series. Abnormal results in order of prevalence were recorded for the HC (18.2%); saccule (14.1%); utricle (8.1%); PC (7.1%); and AC (2.0%). While abnormalities on our tests were not high, they suggest that TBI can potentially affect any part of the labyrinth/nerve. This contrasts with a recent study of 21 chronic post-concussed athletes, where the investigators concluded that concussion mainly affects the inferior vestibular nerve (12). In their study, abnormalities were observed mainly on vHIT and cVEMP with several patients demonstrating combined posterior canal and saccular dysfunction. Others have suggested that TBI is more likely to affect otolith function based on high rates of abnormalities on cVEMP and subjective visual vertical testing of static utricular function recorded during the acute and sub-acute period (29). We observed both patterns of combined posterior canal and saccular dysfunction (Table 2; Figure 2), as well as cases of isolated otolith abnormalities. However, neither pattern was consistent enough to be considered characteristic of chronic TBI.

Different patterns of vestibular end-organ/nerve dysfunction might be expected with different types of injury (36). Occipital impacts are more likely to cause a skull fracture which breaches the otic capsule, causing vestibular dysfunction and other intracranial complications (37). Unfortunately, this clinical detail was not available for our study to determine if there was any relationship. It has also been suggested that injuries that primarily involve angular head acceleration may render the canals more susceptible to injury (38). This contrasts with blast-related injuries which involve shock waves that propagate through the fluid spaces of the body, including the inner ear *via* the CSF and ossicular chain (39). Due to their proximity to the stapes footplate and cochlear oval window, the otolith organs may be particularly vulnerable. This might explain the high rate (52%) of abnormal cVEMP responses in one study of blast-injured patients (13) and suggests that this type of injury might need to be considered separately when considering the effects of TBI on vestibular function. Impact to the side of the skull, particularly if it involves implosive forces through the external auditory canal, might similarly render the otolith organs at risk of injury.

Other identifiable causes of vestibular hypofunction were rare. Two patients had developed spontaneous episodic vertigo, both with a reduced caloric response and normal HC vHIT. This pattern is increasingly recognized as an indicator of Mèniére's disease and has been attributed to the effects of endolymphatic hydrops on the hydrostatic response to caloric stimulation (40, 41). Post-traumatic hydrops, first reported in association with Mèniére-type symptoms by Paparella and Marcini (42), could have a similar effect which might explain the dissociated caloric and vHIT results in some TBI patients. Identification of a patient fulfilling diagnostic criteria for superior semicircular canal dehiscence syndrome (SCDS) (43) aligns with previous studies which suggest SCDS can develop following even minor head trauma (44). Similarly, perilymph fistula identified in association with SBF is in keeping with other studies, indicating that post-traumatic “third windows” are not restricted to the bony labyrinth (34).

Identification of post-traumatic BPPV in our chronic TBI cohort was not unexpected considering the results of previous studies (4, 5, 7). High acceleration-deceleration forces are thought to cause otoconia to detach from the utricular macula, with subsequent relocation into one of the semicircular canals (45). Compared with other causes, post-traumatic BPPV has been associated with higher rates of HC involvement, higher rates of recurrence (46–48) and more intense symptoms (49). In keeping with the reported high recurrence rates, eight patients tested positive for PC BPPV despite documentation of previous particle repositioning. We did not observe any cases of HC involvement, which might suggest that this form of BPPV, once appropriately treated, is less likely to recur.

### Postural stability

Contrasting with tests of peripheral vestibular function, abnormalities on posturography were high (75.5%). Posturography is a functional balance assessment tool which complements the clinical examination and vestibular laboratory testing. Whereas, vestibular testing indicates the function of the vestibular organs and reflex pathways to the eyes and neck, performance on posturography is dependent not only on the integrity of the vestibular input, but how effectively this input is integrated and weighted alongside other sensory input and motor outputs to generate appropriate postural responses in balance tasks of varying difficulty levels. An abnormality affecting any part of the process from the level of the sensory input to the motor output can affect performance on this test.

We found no relationship between vestibular hypofunction and any of the SOT scores. Impaired postural stability was common in patients with and without loss of vestibular function, with both groups demonstrating problems mainly in the more difficult tasks that required high levels of use of vestibular and visual input for balance. Previous studies comparing SOT scores with vestibular test results in head trauma and/or TBI are limited but two studies have similarly shown no relationship between sway patterns and horizontal VOR gain (50), or abnormal SOT scores and cVEMPs or oVEMPs (51). This contrasts with the SOT findings for patients with bilateral vestibulopathy, who show consistent deficits in use of vestibular information for balance (52). As most vestibular deficits in our TBI cohort were unilateral, standing balance will have been influenced more by the degree of vestibular compensation and central adaptation, and potentially other comorbid motor, psychological, cognitive, or medical factors. It is conceivable that these patients would have more difficulty during dynamic balance tasks which involve head movement and thus, stronger vestibular stimulation, which might show a stronger relationship.

Even in the absence of an identifiable vestibular weakness on testing, many patients had difficulty maintaining balance during tasks that required high use of vestibular input for balance. There are several potential explanations for this. On the one hand, some peripheral vestibular abnormalities could have been missed. For example, we cannot exclude a weakness in low frequency lateral semicircular canal function since rotational chair testing was not available. Caloric and VEMP testing are also prone to high inter-subject variability. These tests are useful in identifying unilateral abnormalities but may be less helpful in bilateral lesions, particularly for VEMPs, where the range of normal amplitudes extends down to the noise floor (53). We are also unable to discount the possibility that abnormalities present during the acute phase, which could have triggered maladaptive central compensation, may have recovered.

Of relevance to TBI, is the recent suggestion that chronic imbalance may be caused by selective loss of otoconia, without concomitant loss of hair cells or primary afferents (54, 55). Such pathology might not be detected using VEMPs, which depend mainly on the loosely tethered type I hair cells in the striola region of the macula (56). Alternate clinical measures of utricular function such as ocular counter-rolling or the subjective visual vertical/horizontal during head tilt provide a means of probing the function of the type II hair cells and regular discharging afferents sensitive to static forces, which could be useful in detecting changes in otoconial mass. These tests have only recently been included in commercial VNG systems but provide an avenue for future research in TBI.

An alternative explanation for the balance deficits in our patients is that they represent a problem with the central processing of vestibular and other sensory information. The fact that central oculomotor abnormalities were also predictive of poor balance further supports a central cause and has several interpretations. Firstly, a direct causal relationship due to destabilized vision is unlikely, since the association was mainly with vestibular SOT scores which are based on balance with eyes closed. An alternative possibility is that for some patients, the TBI may have affected brain regions involved in both eye movements and balance. The prefrontal cortex, in particular, is important for attention, multisensory integration, executive function and motor planning of eye, trunk and limb movements (57, 58). A third interpretation for the association between oculomotor function and balance is that it represents more diffuse injury, impacting multiple neural pathways and networks involved in different cognitive, sensory and motor processes.

Only recently have studies begun to investigate the central vestibular pathways which might be involved in post-traumatic dizziness and imbalance. Dysfunction of the brainstem/cerebellar velocity storage mechanism was demonstrated in one study (32); patients with chronic TBI and visually induced dizziness had prolonged time constants and increased slow cumulative eye position on recordings of optokinetic after nystagmus (32). In another study involving provocative visual motion stimuli, subacute TBI patients with these symptoms showed increased activation in several brain regions including area OP2 of the posterior peri-sylvian region and retro-insular cortex (59), a cortical region implicated in motion perception and integration of visual and vestibular information (60, 61). Thus, dysfunction could be occurring at multiple sub-cortical and cortical levels. Interestingly, a recent study showed that a third of patients with acute TBI and normal peripheral vestibular function have impaired self-motion perception on rotational chair testing, coined “vestibular agnosia” (62); these patients also had difficulty using vestibular input for balance. Whether these perceptual changes persist into the chronic phase of the injury has not yet been investigated. Changes in the responsiveness and functional connectivity between brain regions involved in motion perception could interfere with the weighting and integration of sensory information, in turn affecting motor planning and top-down influences on balance.

### Clinical implications

The assessment of people with TBI can be challenging. Often the effects of fatigue need to be tempered against the desire to complete multiple tests. Appointments may need to be extended or divided over several days to allow for breaks, and there is no clear consensus regarding which tests should be prioritized.

Our results suggest that functional tests of balance are essential for this group of patients. The SOT performed using computerized posturography (or alternative, comparable technology) provides objective data on postural sway. This information provides a baseline for rehabilitation planning and for monitoring interventions. Incorporation of more dynamic balance tasks is further expected to improve the sensitivity of this type of assessment (63, 64). Other tests not included in this audit, such as measures of dynamic visual acuity and symptom provocation during head movement also provide valuable information about visual vestibular interaction and compensation, with implications for rehabilitation. Positional testing remains important given the high prevalence of BPPV in the TBI population and the recent suggestion that positional vertigo could be masked by vestibular agnosia (6, 62).

Despite the lower yield on our measures of semicircular canal and otolith function, these tests are important for defining the topography and severity of any deficit and help ensure abnormalities are not missed. They are also important for litigation purposes. Prioritizing vHIT over caloric testing as a first, or primary, test of horizontal semicircular canal function is recommended as it is less likely to provoke symptoms. Caloric testing should, however, be considered when vHIT is normal and there is risk of dysfunction, particularly if the patient presents with Mèniére-type symptoms. Symptoms of SCDS require diagnostic confirmation with VEMPs and CT imaging.

### Study limitations

Several limitations of this study stem from the retrospective design. The information available for review was sometimes incomplete, which limited the ability to screen for all potential confounding factors and prevented exploration of the relationship between test results, TBI severity, and imaging investigations. It also meant that there was no formal control group, which may have had some influence on the classification of results, particularly for vertical vHIT which is more dependent on examiner technique (22). As relatively new tests, the sensitivity of vHIT and VEMPs to subtle changes in the vestibular periphery is uncertain and we cannot discount the possibility that same patients may have had very mild vestibular dysfunction, undetectable by our methods of assessment. Measurements of catch-up saccades could improve the sensitivity of vHIT, whilst incorporation of alternate utricular function tests could unveil otolith abnormalities not readily detected using VEMPs.

Secondly, there is a referral bias as patients were from a single specialist center for vestibular evaluation and rehabilitation. Patients tended to have a long duration of, possibly more severe, symptoms than might be encountered in community concussion clinics. The possibility that some of these patients may have developed a vestibular deficit independent of their TBI seems unlikely given the temporal association between symptom onset and the injury. However, it cannot be completely ruled out. Thirdly, vestibular test data were collected by four audiologists who might differ in their approach to dealing with patient fatigue, anxiety and compliance to testing. These factors have the potential to influence the completeness and quality of the test results obtained.

Future prospective studies that combine structured symptom characterization with electrophysiological and perceptual vestibular testing are needed to inform a deeper understanding of dizziness and imbalance in chronic TBI. These investigations should be complemented by functional brain imaging. Re-evaluating patients at different time points, beginning in the acute phase, would further provide insight into the evolution or resolution of dysfunction and the effects of interventions. Investigation of the spatio-temporal characteristics of postural sway such as velocity, frequency and directional properties could uncover specific sway patterns which might differentiate between different mechanisms of dizziness and imbalance. Balance performance during dynamic tasks such as walking and running, measured with instrumented technologies rather than clinician-judged performance, may add to the understanding of balance in more challenging situations.

### Conclusions

Dizziness and imbalance in chronic TBI were associated with impaired postural stability, particularly for tasks that required high levels of vestibular and visual input for balance. Permanent loss of peripheral vestibular function identified through vHIT, VEMP and caloric testing was recorded, but was less common, except when the injury involved a fractured skull-base. There was no specific pattern of end-organ or nerve involvement that characterized this group of patients.

## Data availability statement

The raw data supporting the conclusions of this article will be made available by the authors, without undue reservation.

## Ethics statement

The studies involving human participants were reviewed and approved by the University of Auckland Human Participants Ethics Committee (Ref 024510). Written informed consent for participation was not required for this study in accordance with the national legislation and the institutional requirements.

## Author contributions

RT reviewed results and extracted relevant data for vestibular and oculomotor function testing, performed the statistical analysis, and wrote the manuscript draft. RT and KW prepared the figures and undertook the screening of clinical records. SC reviewed and extracted posturography data for the SOT. KW, DT, PT, and SC reviewed and helped revise the manuscript draft and revisions. All authors contributed to the article and approved the submitted version.

## Funding

RT received support from an Aotearoa Fellowship funded by the University of Auckland Centre for Brain Research.

## Conflict of interest

Author DT is a Co-Director of the New Zealand Dizziness and Balance Centre (NZDBC). Authors RT and KW are either currently, or have previously been employed by the NZDBC to assess patients. The remaining authors declare that the research was conducted in the absence of any commercial or financial relationships that could be construed as a potential conflict of interest.

## Publisher's note

All claims expressed in this article are solely those of the authors and do not necessarily represent those of their affiliated organizations, or those of the publisher, the editors and the reviewers. Any product that may be evaluated in this article, or claim that may be made by its manufacturer, is not guaranteed or endorsed by the publisher.

## Footnotes

^1^Falls as a cause of TBI were defined as an injury resulting from the head striking the ground/floor from a height during standing or walking. This included trips, slips and falls from walls, ladders and other surfaces.
