# Supplementary material for: Patterns of vestibular dysfunction in chronic traumatic brain injury
Source: Front Neurol. 2022 Dec 1;13:942349. doi: 10.3389/fneur.2022.942349 (PMC9751886; doi:10.3389/fneur.2022.942349)
Supplement: Supplementary file 1 [file Data_Sheet_1.docx]

Supplementary Material

**Supplementary File 1: Methods for recording Vestibular Evoked Myogenic Potentials (VEMPs)**

This file outlines the VEMP methodology and protocols which were used at the time of the data review period from January 2015 to December 2019.

**Electrode configuration**

VEMPs were recorded using Ag/AgCl disposable electrodes (Positrace® RTL, ConMed Corporation, USA) and snap leads. NuPrep^TM^ skin preparation gel was used to cleanse the area of skin where the electrodes were placed. For cVEMPs, an inverting electrode was placed over the sternocleidomastoid (SCM) muscle belly, referred to a non-inverting electrode placed over the medial clavicle. For oVEMPs the inverting electrode was placed directly beneath the lower lid margin of the eye, slightly lateral to the pupil. The non-inverting electrode was placed as close as possible, but without touching, on the cheek below. The ground electrode was placed on the sternum. Inhibitory responses (positive surface potentials) appear as a trough and excitatory responses (negative surface potentials) as a peak.

For cVEMPs, an additional electrode placed as close as possible to the first inverting electrode created an extra recording channel which was used to monitor tonic electromyographic activity (EMG) from -10 ms pre-stimulus to 70 ms post-stimulus onset. Biofeedback was provided to patients to help maintain SCM activation at a relatively consistent and equal level (across left and right ear trials) throughout the recording. The system was set to only accept trials where the average level of tonic EMG fell between 70 and 130 microvolts.

**Stimulus and recording parameters**

| Stimulus modality | Transducer | Stimulus  polarity | Stimulus  intensity | Stimulus duration  (rise-plateau-fall) | Stimulus envelope |
| --- | --- | --- | --- | --- | --- |
| AC | ER 3A insert earphones | Condensation | 125 dB SPL | 6 ms  (2-2-2) | Blackman |
| BC | B&K minishaker | Condensation | Driving voltage (24V) | 4 ms  (2-0-2) | Blackman |

*BC stimulation involved amplification through an external amplifier (Bruel and Kjaer, model 2718)

| Reflex Pathway | Averages | Filter settings  (Hz) | Time  window (ms) |
| --- | --- | --- | --- |
| cVEMP | 125 | 20 to 2000 | -10 to 70 |
| oVEMP | 60 | 1 to 1000 | -10 to 70 |

For cVEMPs, reflexes were recorded during head-elevation from the semi-recumbent position (torso elevated at 30°above horizontal). Where muscle activation was insufficient, the patient was instructed to turn their head slightly in the direction opposite the sound until the EMG activity fell within the desired range. The clinic normal range of normalized amplitudes for AC sound using this protocol was 0.28 to 2.25 (n=22; aged 21 to 58).

For oVEMP recordings, participants were instructed to look up as high as possible until the stimulus stopped. The normal range of BC oVEMP amplitudes for the clinic (n=24; aged 21 to 53) was 3.5 to 25.8µV.

Stimuli were presented at a rate of 5.1/second. Two trials of stimuli were presented for each reflex.

**Analysis**

For each reflex, traces for the two trials were checked for reproducibility then added to obtain a grand-averaged waveform. Peak-to-peak amplitudes were calculated from markers positioned over the corresponding peaks and troughs of the grand-averaged waveforms. Amplitude asymmetry ratios were then calculated using the Jongkees formula. For cVEMPs, this was performed on normalized amplitudes to account for subtle differences in tonic muscle activation.

**Middle ear abnormalities and protocol adaptations**

To control for the influence of middle ear pathology on cVEMPs, all patients are first assessed with otoscopy and tympanometry and sometimes, acoustic reflex testing and audiometry. Bone-conduction cVEMPs were included as a cross-check in the event of an abnormal finding on these tests (i.e., negative middle ear pressure < -100 daPa, immobile tympanic membrane, tympanic membrane perforation, ossicular discontinuity or fixation, air-bone gap > 10 dB on audiometry).

Thirteen patients included in the retrospective review had either unilateral or bilateral middle ear abnormalities, requiring additional bone-conduction testing. Bone-conduction, which is a more robust stimulus, was also used for people over the age of 60 with absent responses to AC sound (n=2 patients in this study) since absent reflexes are known to occur in older healthy control subjects. Another patient was tested with bone-conduction instead of air-conduction due to severe hyperacusis.
